# Dental Surgery as Applied in the Armies of the Late Confederate States

**Published:** 1867-08

**Authors:** W. Leigh Burton

**Affiliations:** Dentist, Richmond, Va.


### ARTICLE IV.

### *Dental Surgery as applied in the Armies of the late Confederate States.*

BY W. LEIGH BURTON, Dentist, Richmond, Va.

DENTAL SURGERY has assumed such importance within the last few years as to place it much above the position which was at one time assigned it, when barbers and blacksmiths were about the only representatives of the profession. Concerning the primitive condition of the art in this city, the late, venerable Samuel Mordecai in his delightful book, *Richmond in bygone days*, says:

"Now adays the profession of dentistry gives lucrative employment in our city to a score of practitioners. In the days of my boyhood, only one *Tooth-drawer*, who probably never heard the word dentist, did all the work and all the mischief in the dental line."

"Peter Hawkins was a tall, raw-boned, very black negro, who rode a raw-boned, black horse, for his practice was too extensive to be managed on foot, and he carried all his instruments, consisting of two or three pullikins in his pocket. His dexterity was such, that he has been known to be stopped in the street by one of his distressed brethren, (for he was of the church,) and to relieve him of the offending tooth, gratuitously, without dismounting from his horse. His strength of wrist was such, that he would infallibly extract, or break a tooth, whether the right or wrong one. I speak from sad experience, for he extracted two for me, a sound and an aching one, with one wrench of his instrument."

"On Sundays he mounted the pulpit instead of black bare bones, and as a preacher he drew the fangs of Satan with his spiritual pullikins, almost as skillfully as he did the teeth of his brother sinners on week days, with his metallic ones."

It is undeniably the case that, even in this period of the world's progress, there are some persons who profess to have but little faith in dentistry and who boast of never having required dental operations; but it is generally found that they are blessed with very sound teeth and consequently, would have about as much occasion to employ a dentist as a perfectly healthy man would in calling in a physician. And stranger still, there are actually *physicians* who are not only skeptical of dentistry and regard it as a "modern innovation," but even look upon its practitioners as upstarts, and as aspirants for titles and honors which belong only to themselves. It is perhaps unnecessary to say that, generally speaking, those who are so jealous of an encroachment upon their rights and privileges are either very old men who have followed the same beaten track, and prescribed the same medicines for the last half century; or else very young ones with little or no brains, whose appreciation of a diploma is based upon the difficulties and length of time in acquiring one, extending generally over a considerable period. In spite, however, of the prejudices of some, it is true, very few narrow minds, society at large appreciates the importance of dental surgery as bearing upon the health and comfort of every community, and as a rule, its practitioners keep pace with the age in education, refinement and accomplishments. Every year witnesses the advent of candidates for public favor whose training in our colleges has been careful and complete, the introduction of some new invention or the application of some new principle; and it is with some pride that we compare the dentistry of to-day with that of fifty years ago.

During the progress of the late unhappy war, it soon became apparent that the soldiers of the Confederate armies stood sadly in need of the services of dentists. Most of them being from extreme sections of the country, without means, being cut off from all communications with their homes, and their pay being totally inadequate to meet the most ordinary and pressing wants, it was out of the question for them to attempt to pay for dental operations. Particularly when it is remembered the price of one gold filling in the depreciated currency of the Confederacy, was more than six months pay of a private! The price demanded for gold foil in 1864 and the beginning of 1865 was sixty-four dollars per oz. in gold coin. This amount in confederate money would be, three thousand eight hundred and forty dollars, for the prevailing price of gold was sixty for one. Having to pay so enormously for materials—the value being enhanced from the fact of their having to run the blockade—the charges of dentists were proportionally high. The charge for a gold filling was $120.00, for extracting a tooth $20.00, and for an upper set of teeth on gold or vulcanite base, from $1800.00 to $4000.00. Let it not be understood that high prices were confined to dentistry alone. It was no uncommon thing to pay $1800.00 for a coat, $300.00 for shoes, $1000. 00 for cavalry boots, and from $300.00 to $500.00 for an ordinary felt hat. A man considered himself lucky in being able to purchase a turkey for $300.00, which was indeed above the gold standard, but confederate money was plentiful and turkeys were scarce, and the scarcity became greater in proportion to the frequency of the raids of Federal commanders, so that before the close of the war it was with difficulty that fowls could be obtained for love or money.

From the above examples of prevailing prices it is very clear that a confederate soldier getting eighteen dollars per month could not afford to pay for dental operations, and the question arose, what was to be done for the amelioration of his condition? This question was solved by the rigid conscription laws passed by Congress; dentists were conscripted, and the Surgeon General of the late Confederate States, with a forethought and humanity characteristic of the man, determined at once to make their valuable services available to the armies.

It must not be imagined that dentists were conscripted without a show of resistance on their part. Physicians of "seven years practice" were exempted from military service, and as dentists were not mentioned, it was contended by some that Congress intended to include them as *special practitioners.* This interpretation of the law met with not only the most violent opposition from some few old fogy physicians, who ridiculed such assumptions, but was overruled by the war department through its representative Judge Campbell. and the conscription of dentists was ordered to be proceeded with at once.

Already eminent legal opinion had sustained the dentists that they were exempt by the law as *physicians,** but to test a matter in which so many were interested, Dr. Hunter of North Carolina, brought his case before the courts of his State, and Judge Pearson decided that *he was exempt.* Congress, having heard of the decision in this case, upon revising the exemption laws, determined that no ambiguity of language should lead to any further exemption of dentists. They seemed to have forgotten in consulting the requirements of every community, that the same efficiency could not be reasonably expected in dentists over the conscript age as in younger men. It does not follow that the older a man is the better the dentist. Good eyesight and a steady hand are indispensible. This enlightened body, however, not looking upon the matter in the same light, had inserted in the clause exempting physicians, "the term physicians *not to include* dentists," and thus the question was settled.

Surgeon General Moore immediately made application to the Bureau of Conscription for the detail of dentists for duty in the hospitals, and after some months of experiment, he caused to be issued the following order.

"OFFICE OF THE MEDICAL DIRECTOR<br>C. S. HOSPITALS IN VIRGINIA,<br>RICHMOND, *Va., Nov.* 4. 1864.

CIRCULAR<br>*No.* 15.

I. As far as practicable in future, the operations of dentistry required

*Reference is made particularly to the late Gen. Geo. W. Randolph Sec'y War of the C. S.

in General Hospitals in Virginia, will be performed by officers, soldiers or conscripts assigned to those duties, who are dentists by profession.

II. Examinations will be made, at such times as may be fixed by the surgeon in charge, of each officer and soldier admitted into hospitals, and the necessary operation performed with the concurrence of the attending Medical officer.

III. Dentists are expected to be provided with their own instruments, but the necessary materials and files will be purchased with the hospital funds, and requisition made for other instruments thought necessary.

IV. Dentists will have the rank, pay and perquisites which their position in the army entitles them, and in addition, such extra duty pay for extraordinary skill and industry, as the Surgeon General will allow, in accordance with general order, No. 66, A. & I. G., office, current series.

V. Monthly reports of Dental operations and accompanying registers in accordance with forms furnished, will be forwarded through the Surgeon in charge and through this office, to the Surgeon General by the 5th of the month succeeding.

(Signed) WM. A. CARRINGTON,

Medical Director of Gen. Hospitals in Virginia.

It would be next to impossible to form an idea of the wretched condition of the teeth of Confederate soldiers. The great majority had been several years in service without as much as even having had their teeth examined ; and this neglect coupled with habits of carelessness, an absence of tooth brushes, and the miserable and scanty food upon which they subsisted, only hastened their destruction.

In selecting a material with which to fill the teeth of these men, it was desirable to have something which was not costly, that could be easily applied, and which would at any rate, preserve them until such a time when gold might be substituted, and for this reason amalgam was chosen.

Without stopping to discuss the merits or demerits of this material, it is enough to say that, for this purpose, it answered admirably. In every case the carious portion of the tooth was thoroughly removed, and when a large number of teeth required attention, the operations were greatly facilitated by the use of it.

The principal operations performed were filling, extracting teeth and removing the tartar, which from neglect, had accumulated in nearly every mouth to a most extraordinary extent ; the adjustment of fractures of the bones of the mouth and the treatment of wounds of the face. From the time of the assignment of the first dentist to duty in March 1864, the number of operations performed exceeds all belief. A day's work consisted of from twenty to thirty fillings, the preparation of the cavities included, the extraction of fifteen or twenty teeth, and the removal of the tartar *ad libitum !*

Besides a detailed monthly report of each operation performed, in which the date, patient's name, rank, regiment, company, and the operations, were required to be given, a summary was attached in which they were consolidated after the following order: Number of patients operated on—Teeth extracted—Fillings inserted—Teeth cleaned of tartar—Fractures adjusted—Other operations—Total number of operations performed.

Concerning fractures of the bones of the mouth, the number of cases was not so large as might have been expected from the fact that, in the year 1864 to which reference is made—the fighting was in a great measure from behind breastworks, when of course the head is more exposed than the rest of the body. In such cases as presented themselves in and around Richmond, the reduction of the majority of them was effected through the agency of *gutta percha.* The case of Jas. H. Hutchinson, Co. C., 53d Georgia regiment, 2nd division, Jackson Hospital, affords an example. His was a fracture of the superior maxilla, left side, involving the first and second bicuspid teeth. After forcing the fracture into its proper position, gutta percha—having been softened in warm water—was pressed on the teeth included in the fractured portion and extended to the firm teeth, the lower jaw closed and the teeth embedded in the lower portion. It was then carefully removed, placed in cold water to harden and readjusted afterwards. This accomplished all that could be desired. The fracture was held perfectly firm, the material afforded a pleasant rest to the jaw, and left an opening through which food might be received, and at the same time, it was not affected by the secretions of the mouth, or by discharges from the wound.

The case of Lieut. Morehead—North Carolina Cavalry, quartered in general hospital No. 10, was similar to the above, only more complicated. A minnie ball carried away the canine, first and second bicuspid teeth, left side, inferior maxilla, fracturing the bone to the left of the *symphysis*, passed under the tongue, the counter stroke fracturing the right side of the bone near the *mental foramen*, and passed out under the angle of the *ramus*, carrying fragments of teeth and bone with it. Bond says in his work on dental medicine, Article Fractures; "The general treatment of fractures consists in meeting the following indications; 1st. To restore the displaced pieces of bone to their natural position. 2d. To keep them there; and 3d. To afford any additional aid which the nature of the injury and the constitutional circumstances may require." The two first indications were fully met in this case, but to ensure certainty, after the *interdental* splint had been applied, an outer splint of the same material was made to conform to the shape of the jaw and the common bandage applied. The next day the patient feeling so entirely free from pain, he was furloughed to go home, where he soon recovered. The only thing which the writer of this article claims for the use of gutta percha in adjusting fractures of the jaw bones, is, its originality as far as concerns himself. Pasteboard softened with vinegar and water has been the usual material employed in such cases; but it appears to the writer that any man possessed of common sense and knowing the peculiarities of gutta percha, would naturally fix upon this substance. Dr. James Bolton in a communication to the *Richmond Medical and Surgical Journal,* states that he has used it in cases of fractures of the jaw bones, and no doubt others have with the same success.

The interdental splint of Dr. Bean of Atlanta, Ga., upon which an elaborate article was furnished the January number of the journal above referred to, by Dr. Covey, deserves especial mention. But from the subjoined letter even this appears to be nothing new. The writer having having heard of the accident to Mr. Seward in April, 1865, by which he sustained a fracture of the lower jaw, was emboldened by the dictates of humanity to call the attention of the Surgeon General of the United States to this splint, and received the following reply.

"SURGEON GENERAL'S OFFICE,
WASHINGTON CITY, D. C.,
*April* 26, 1865.

SIR:—

I am directed by the Surgeon General to acknowledge the receipt of your letter of the 24th inst., calling his attention to an interdental splint (Dr. Bean's) as likely to be of service in Mr. Seward's case of fracture of the jaw, and to inform you that the same splint is now made by Mr. Gunning in New York.

Very respectfully your obedient servant,
By order of the Surgeon General.
(Signed,) C. H. CRANE,
*Surgeon U. S. Army.*

While this reply disposes quite summarily of Dr. Bean's interdental splint, it is questionable if Mr. Gunning *was* making precisely the *same* splint, though it might have been constructed on the same general principles.

During the occupation of Atlanta by the Confederate forces this splint was applied in over *one hundred cases* with such invariable success, that Surgeon General Moore ordered Dr. Bean to Richmond, in order that his splint might be laid before the army Medical Board of the Confederate States. A meeting of the Board having been ordered at the private office of the writer, the drawings and models exhibited and full explanations made by Dr. Bean in person, the members, whilst arguing that the principles of it were not entirely new, were unanimous in recommending its general adoption; and shortly afterwards—dentists on duty having been instructed in its construction—the Surgeon General ordered a ward to be prepared at the Robertson hospital for the exclusive treatment of all cases in which it could be applied. A minute account of this splint having already been published, it is scarcely necessary to enter into a particular description here. Suffice it to say that, models of the parts having been obtained by a peculiar and scientific process, it is constructed of *vulcanized rubber*, instead of *gutta percha*, and applied in the same manner.

Among patients in the hospital, the treatment of exposed pulps was practiced to some extent and many teeth saved which would otherwise have been lost; and whilst the paste is used in the office of nearly every dentist, the proportions are given for the reason that, its application having been attended with such uniform success, some might desire to test its efficacy. They are the following:

| | | |
|---|---|---|
| ℞ | Acid. Arsenios . . . . . | gr. xxx |
| | Morphiæ Sulphatis . . . . | gr. xx |
| | Kreosoti . . . . . . | q. s. |

The insertion of artificial teeth on plates was not included in the operations allowed. In some instances pivot teeth were inserted or readjusted, and in this connection it might not be out of place to mention a plan adopted by the writer for securing a more perfect adaptation of the tooth to the fang. It consisted of a piece of thin rubber introduced between the tooth and fang in the shape of an "O," through which the pivot entered the fang. In readjusting these teeth, it was often the case that the fang after being filed, caused the old tooth to be so much shortened as to be useless, which in a time of great scarcity of materials was no trifling consideration. By the use of the rubber the tooth was not only lengthened sufficiently to be used again but it was found that *moisture was entirely excluded* as long as the pivot remained firm, thereby adding greatly to the preservation of the fang.

The experiment of dental surgery in hospitals having been received with such universal favor, dentists were assigned to the most important points thoroughout the Confederacy; and so convinced was the Bureau of Conscription of its importance, that notwithstanding the need of men for field service was more urgent than ever, no obstacle was nterposed to their appointment. Thousands of men throughout the southern states can attest to the benefits they received by its adoption, and it is confidently believed that had the necessity for its longer continuance existed, it would have led to the establishment of a regular Bereau of Dental Surgery, associated with the medical staff of the army.

Dentists of every land owe a debt of gratitude to a man who gave official recognition to the importance of their profession, and who extended to those under him every encouragement in the prosecution of their arduous duties; and no matter what differences may exist in regard to opinions honestly entertained, or a line of conduct dictated by a sense of duty, they owe more to Samuel Preston Moore, Surgeon General of the late Confederate States army, than to any man of modern times.

